# Production of Valuable Compounds and Bioactive Metabolites from By-Products of Fish Discards Using Chemical Processing, Enzymatic Hydrolysis, and Bacterial Fermentation

**DOI:** 10.3390/md17030139

**Published:** 2019-02-27

**Authors:** José Antonio Vázquez, Araceli Meduíña, Ana I. Durán, Margarita Nogueira, Andrea Fernández-Compás, Ricardo I. Pérez-Martín, Isabel Rodríguez-Amado

**Affiliations:** 1 Grupo de Biotecnología y Bioprocesos Marinos, Instituto de Investigaciones Marinas (IIM-CSIC), C/Eduardo Cabello, 6, CP 36208 Vigo, Galicia, España; araceli@iim.csic.es (A.M.); anais@iim.csic.es (A.I.D.); marga@iim.csic.es (M.N.); afcompas@inidep.edu.ar (A.F.-C.); ricardo@iim.csic.es (R.I.P.-M.); 2 Laboratorio de Reciclado y Valorización de Materiales Residuales (REVAL), Instituto de Investigaciones Marinas (IIM-CSIC), C/Eduardo Cabello, 6, CP 36208 Vigo, Galicia, España; 3 Instituto Nacional de Investigación y Desarrollo Pesquero (INIDEP), Paseo Victoria Ocampo N°1 Escollera Norte, Mar del Plata C.C.175-7600, Argentina; 4 Laboratorio de Bioquímica de Alimentos, Instituto de Investigaciones Marinas (IIM-CSIC), C/Eduardo Cabello, 6, CP 36208 Vigo, Galicia, España; 5 Departamento de Química Analítica y Alimentaria, Universidad de Vigo, Campus As Lagoas s/n, 32004 Ourense, España; sabelara@uvigo.es

**Keywords:** fish discards, by-products valorization, fish protein hydrolysates, bioactivities, marine peptones, lactic acid bacteria

## Abstract

The objective of this report was to investigate the isolation and recovery of different biocompounds and bioproducts from wastes (skins and heads) that were obtained from five species discarded by fishing fleets (megrim, hake, boarfish, grenadier, and Atlantic horse mackerel). Based on chemical treatments, enzymatic hydrolysis, and bacterial fermentation, we have isolated and produced gelatinous solutions, oils that are rich in omega-3, fish protein hydrolysates (FPHs) with antioxidant and antihypertensive activities, and peptones. FPHs showed degrees of hydrolysis higher than 13%, with soluble protein concentrations greater than 27 g/L and in vitro digestibilities superior to 90%. Additionally, amino acids compositions were always valuable and bioactivities were, in some cases, remarkable. Peptones that were obtained from FPHs of skin and the heads were demonstrated to be a viable alternative to expensive commercial ones indicated for the production of biomass, lactic acid, and pediocin SA-1 from *Pediococcus acidilactici*.

## 1. Introduction

In recent years, the worldwide capture of fish from fishing activities has exceeded 150 million tons [1]. From these, a huge amount of material (more than 25%, but in some cases up to 70%) is considered as by-product (skeletons, viscera, heads, etc.) after human food processing. These wastes must be well managed to avoid environmental problems and to try to maintain resource sustainability [2]. In addition to these large volumes, fish by-products that are generated through canning and freezing activities must be included, as well as the new fish discards biomasses that will be generated in fulfilling the main goals of the Landing Obligation of the European Commission (EU) Common Fisheries Policy [3]. From 2019, the Landing Obligation will force all fishing vessels to keep and not discard all of the species that are caught that are subjected to quota or have a minimum legal size, as well as underutilized commercial species. That is why valorisation solutions have to be implemented to manage this new situation while using available and simple technological alternatives that do not have high associated operating costs [4,5].

The joint production of fishmeal and oils is the most common utilisation of fish by-products, including fish discards origin, but it is not the most valuable and sustainable when a fishmeal plant is not located nearby. Different strategies have been proposed in order to deal with the new biomasses that will be generated from 2019. The valorisation alternatives depend on the fish species and the reasons for discarding: a) fish under minimum conservation reference size species, b) non-quota fish species, and c) fish species with low commercial or interest value. In this last case, fish specimens can be used to develop new products for direct human consumption [3]. After heading, gutting, and the mechanical separation of muscle from skins and bones, minced muscle is an excellent raw material for the elaboration of several seafoods formulations [6,7]. The corresponding by-products that are generated from this approach, heads, and the mixture of skins and bones, could be specifically treated by chemical, enzymatic, and/or microbial processing [8,9,10] to produce different valuable biocompounds that are useful as materials for nutraceutical, food and biotechnological applications. However, similar integral alternatives have not yet been explored for wastes from discarded fish species.

In this context, the present study is the first time that by-products of skin and heads from discarded fish species in trawler fisheries in North-West Spain (megrim, boarfish, hake, grenadier, and Atlantic horse mackerel) have been evaluated for the production of gelatins, oils, fish protein hydrolysates, bioactive peptides, and marine peptones that are useful as nitrogen sources for microbial productions.

## 2. Results and Discussion

### 2.1. Gelatin Isolation from Skin and Bones (SB)

Samples of skin and bones (SB) from the five fish discards were processed using the protocol for the optimal isolation of gelatins from skin wastes of tuna, Greenland halibut, and blue shark [10]. The results of gelatin extraction yield, content of proline plus hydroxyproline, and the strength of gels are summarised in Table 1. SB from grenadier did not yield gelatinous solution, and in other cases, the yields were not too remarkable (1.7% *w*/*w* of SB for Ha as the best option) when they are compared with gelatin that is recovered from shark and tuna wastes (more than 12% *w*/*w*). Our yields for isolated megrim (Me) and hake (Ha) gelatins were quite lower in comparison to the data reported (10% *w*/*w* of skin) by Montero and Gómez-Guillén [11] while employing a protocol that was based on thermal treatment of collagen previously extracted by combining saline, alkalis, and acetic acid with clean skins. However, using these last steps, a lower amount of gelatins from megrim (7.4% *w*/*w* of skin) and hake (6.5% *w*/*w* of skin) were recovered by the same authors [12]. The differences in yields that were found in the present work could be due to the method of extraction, which is optimal for tuna and blue shark skins, but is perhaps too aggressive for Me and Ha skins, together with the type and state of the substrate. In fact, our results were calculated based on the SB weight, and SB were mechanically obtained (in a very aggressive way) and then processed without being previously cut into small portions and without an initial wash in saline solution.

The values of Pro + OHPro, higher than 16%, are in line with those that were expected for gelatin solutions [13,14]. In this context, the percentage of gelatin that was obtained in the current work was similar to the results reported for the gelatin extracted from sole, squid, megrim, and hake [12]. Regarding gel strength analysis, only gelling samples that were obtained from Ha and Me showed certain firmness to the penetration of the cylinder probe of the texture analyser. The values of blooms for both gelatins were significantly lower than those observed for gelatin that was obtained from collagen extracted from mackerel skin [14]. Moreover, gels that were prepared with megrim and hake gelatins also isolated from skin collagen were much harder than our extracts [11,12].

### 2.2. Production and Chemical Composition of Fish Protein Hydrolysates (FPHs) and Oils Recovered

Table 2 shows the balance of products that were obtained after Alcalase hydrolysis of SB and head (H) substrates as well as the approximate composition of FPHs. The amount of insoluble material isolated from FPHs processing was higher than 18% in SB and 13% in H. The maximum percentage for bones was found in SB_Bo and SB_G samples, and SB was always a superior bone source than H (*p* < 0.05). The capacity of Alcalase for the digestion/liquefaction (V_dig_) of SB and H was, in all cases, larger than 85% (H_Me), with a maximum value of 91% being obtained in SB_G. No clear differences were detected between V_dig_ data of SB and H. In half of the samples (SB_G, SB_H, H_G, H_Ha, and H_Me), the recovery of fish oil after proteolysis was ineffective, but in heads of HM, a 2.4% *v*/*w* of oil was extracted (Table 2). Regarding the profile of fatty acids that were present in fish oils, the most abundant were always oleic (12.6–15.4%) and palmitic acids (16.1–19.4%), followed by docosahexaenoic acid (DHA) (8.6–16%) and eicosapentaenoic acid (EPA) (4.1–10.1%) (Table S1, Supplementary Materials). Because the amount of oil that was obtained from SB_Bo was very low (0.2% *v*/*w*), no composition of fatty acid was determined. The sum of essential omega-3 (DHA+EPA) was greater than 12.8% (26.1% in SB_Me), being 16.7% for SB_HM and 20.2% for H_HM. These results are in agreement with values that were reported for oil obtained from fillets of Atlantic horse mackerel (19.5–22%) and individuals of horse mackerel from Mediterranean Sea (22.1%) [15,16,17]. However, the percentage of DHA and EPA in oil from SB_Me was much higher than oil extracted from megrim liver [18]. Omega-3/omega-6 ratios ranged from 2.2 to 5.8, demonstrating its beneficial composition for nutraceutical formulations [19,20].

As it can be observed in Table 2 and Table S2 (Supplementary Materials), the values of total protein (Prs, Pr-tN, and Pr (Σaa)) from FPHs that are produced from SB were in all situations higher than that found for H (*p* < 0.05). SB_G and SB_Me were the hydrolysates to yield the highest protein content. The differences between protein data that were quantified by the three methods were lower than 16%, in many cases being inferior to 2%. Such small discrepancies are due to the fact that Prs only determines soluble protein and Pr-tN also measures particulate protein. The results of FPHs digestibilities (Dig) were always higher than 90% (94% for SB_Me). The amino acids profile is a fundamental parameter to verify the validity of FPHs for human nutrient uses [21]. In all FPHs, essential amino acids (Val, Lys, Met, Ile, Leu, His, Arg, Phe, and Thr) are present in good proportion, although the most predominant are aspartic and glutamic acids. Based on these composition of amino acids, together with the excellent values of Dig, we can indicate that present FPHs have a valuable and potential application in pet and aquaculture feed as substitutive of fish meal [22,23], nutritive broths for microbial productions [24], and in human food supplements [25,26]. To the best characterization of FPHs, total sugars were also determined, with ranging values from 0.50 to 1.15 g/L, being generally the content lower in hydrolysates from SB than those that were obtained from H.

**Figure 1:**
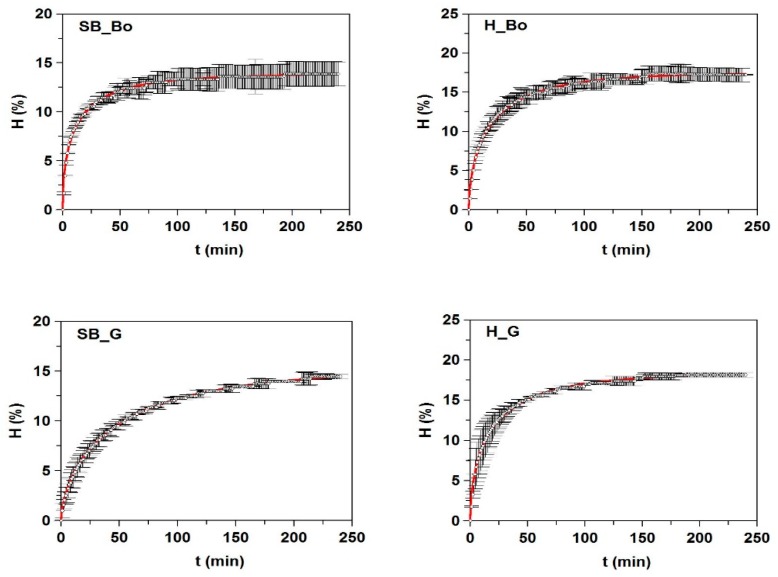

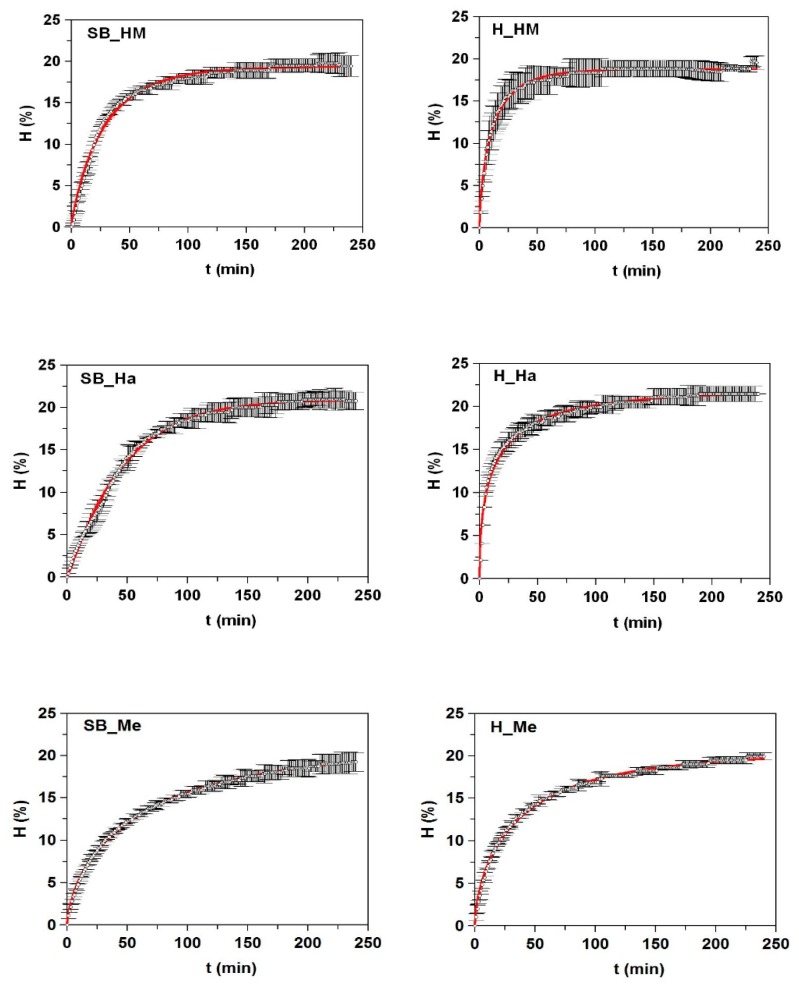
Kinetics of skin/bone (SB) and head (H) hydrolysis from fish discards using Alcalase. The experimental data (symbols) were fitted to the Weibull Equation (1) (continuous line).

All kinetic data of hydrolysis were accurately described by mathematical model (1) and were statistically confirmed by the values of R^2^ > 0.989, the consistency of fittings (*p*-values < 0.005), and the significance of the parameters for α = 0.05 (Figure 1 and Table 3). The maximum degrees of hydrolysis (*H_m_*) were generated in FPHs from heads and they were slightly higher in H_Ha and H_HM (21.9% and 21.4%, respectively). The value of *H_m_* from H_Bo (17.6%) was similar to the final degree of hydrolysis at 24 h (17%) obtained by papain digestion of boarfish individuals [27]. The values of *τ* were lower in FPHs (mainly from heads) with higher *H_m_* values and higher values of *v_m_* (faster Alcalase hydrolysis). The variations in degree of hydrolysis that were observed between FPHs may be due to differences in the type and molecular structure of the proteins from each fish by-product, since the experimental conditions of hydrolysis were equal for all cases and the amino acids composition was very similar in the hydrolysates (Table S2, Supplementary Materials).

### 2.3. In vitro Bioactivities of FPHs from Fish Discard By-Products

Table 4 shows the values of the antioxidant and antihypertensive activities that were determined for the FPHs samples generated at 4 h of Alcalase hydrolysis. Overall, the results of antioxidant activity were not especially significant. For instance, the percentages of 1,1-Diphenyl-2-picryhydrazyl (DPPH) were less than 50%, with only H_G and H_Bo reaching greater than 40%, whereas the SB_Bo data were negligible. Crocin and 2,2′-azinobis-3-ethyl-benzothiazoline-6-sulphonic acid (ABTS) quantifications confirmed these results. All of these values are low in comparison to the antioxidant activity produced by other marine and fish peptides and hydrolysates [8,28,29,30,31,32]. Nevertheless, FPHs from Pacific hake (*Merluccius productus*) showed activities that were in the same range (18–30%) as SB_Ha and H_Ha [33]. Moreover, ABTS and Crocin data were also in line with the antioxidant results for hydrolysates from red scorpionfish and blue whiting [34,35].

The percentages of antihypertensive inhibition (*I_ACE_*) were greater than 7%, with a maximum response in H_Bo. The large majority of *I_ACE_* are in the range of 25.4% and 73.8%. However, the samples of SB_Bo and H_Me did not show activity. Hydrolysates from SB were higher, but differences between the different origins of by-products were not statistically significant. Our best FPH (SB_Bo = 74%) led to higher inhibition than referenced for papain-FPH (~45%) and alcalase-FPH (~65–70%) from complete boarfish wastes [27]. The results from SB_Ha and H_Ha were similar to those that were proposed for Pacific hake fillet hydrolysates generated with protamex [36] and FPH from Cape fish sawdust and cutoffs [37]. However, the bioactivities of HM samples were inferior to those previously reported for horse mackerel processed with trypsin [17]. Dose-response bioassays for obtaining IC_50_ values of hydrolysates were only performed in the samples with *I_ACE_* >50%. In terms of this parameter, H_Bo showed the strongest activity (178 μg/mL) and had the lowest value of IC_50_. This data was in concordance with those found in FPH from Pacific hake (165 μg/mL) [36], but were much stronger than those obtained for European hake heads (260 μg/mL) [21], red scorpionfish muscle (970 μg/mL) [34], head of red scorpionfish (490 μg/mL) [38], and blue whiting fillets (1.34 mg/mL) [39].

### 2.4. Low-Cost Media with Peptones from FPHs for Pediococcus acidilactici Culture

Peptones are defined as the water soluble mixture of proteins, peptides, free amino acids, and small amounts of nucleotides and carbohydrates that are not coagulable by heat, and are obtained by the hydrolysis (thermal, chemical, or enzymatic) of animal, vegetal, or microbial substrates. They are the most important and expensive source of organic nitrogen in the commercial media for the cultivation of microorganisms [40,41,42]. Therefore, inexpensive sources of peptones from food wastes are in high demand [43]. Our fish peptones from FPHs can be an adequate ingredient of bacterial growth media and *P. acidilactici* is an ideal candidate for the evaluation of their nutritive validity [44,45].

**Figure 2:**
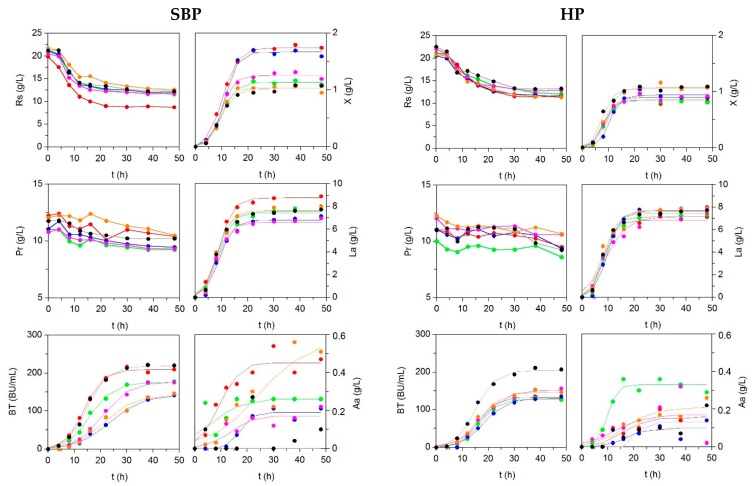
Culture kinetics of *P. acidilactici* grown on different media formulated with peptones obtained from skin/bone (SBP, left) and head (HP, right) by-products from fish discards. MRS medium was used as control. 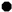: SBP_G and H_G; 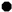: SBP_Ha and H_Ha; 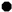: SBP_Bo and H_Bo; 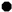: SBP_HM and H_HM; 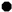: SBP_Me and H_Me; 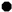: MRS. Experimental data of biomass (X), lactic acid (La), acetic acid (Aa), and pediocin (BT) were fitted to the Equation (2). Reducing sugars (Rs) and protein (Pr) uptakes were also shown. The confidence intervals of experimental data (for two replicates) were in all cases less than 10% of the experimental mean value and omitted for clarity.

Low-cost broths were formulated, substituting the commercial peptones that are present in MRS (meat extract and bactopeptone) by fish peptones, but maintaining the same level of soluble protein as commercial ones (Table S3, Supplementary Materials). Figure 2 and Table 5 illustrates experimental kinetics of biomass, organic acids, and in productions, together with nutrient uptakes in all media tested (including MRS as control). The production of *P. acidilactici* biomass in alternative media was similar or higher than observed in MRS. Experimental sigmoid profiles of the productions were accurately modelled by logistic equation (2), with coefficients of determination that are higher than 0.968 and *p* < 0.001 in all cases. The productions of acetic acid were lower than 0.6 g/L and kinetics were something more random, but also simulated by logistic equation.

The maximal growths (defined by means of *H_m_* parameter) were obtained in the cultures that were formulated with SBP (less in the case of SBP_Me). The values of *H_m_* in SBP_G and SBP_Bo were significantly greater than the rest of media. Nevertheless, the values of lag growth phases and maximum growth rates were similar in all media (*p* > 0.05). The most efficient peptones in producing biomass in terms of the growth yields regarding nutrient uptakes (*Y_X/RS_* and *Y_X/Pr_*) were also SBP_G and SBP_Bo. These findings are in agreement with the results that were reported for the growth of lactic acid bacteria using nitrogen sources obtained from enzymatic and alkaline effluents that are generated in the isolation of chitin from squid pens [46].

Regarding lactic acid, the highest and lowest *La_m_* values were observed in media with peptones from skins/bones of grenadier and horse mackerel, respectively. Maximum lactic acid formation in SBP_G was significantly higher than MRS (*p* < 0.05). The lag phases and maximum rates of lactic acid productions were statistically identical for all peptones evaluated (*p* > 0.05). SBP_Me was the most efficient peptone for the formation of lactic acid per glucose consumption and HP_HM showed the highest value of *Y_La/Pr_*. The production of pediocin, in terms of *BT_m_* values, was similar in SBP_G and MRS (*p* > 0.05) and much greater than in the rest of broths. Maximal rates of pediocin production were found in these cases, with MRS being the most productive and effective nutrient formulation for pediocin production, followed by SBP_HM, SBP_HA, and SBP_G.

The outcomes of this study are in line with other studies of marine peptones that are derived from viscera by-products of several fish species, which also addressed their validity as proteic nutrient in culture media to produce bacteriocins from lactic acid bacteria [42,47,48,49]. From an economical point of view and based on the commercial prices of MRS ingredients, the new fish peptones led to an important reduction of ingredient costs in the 3–4.5-fold range for biomass production, 2.5–3-fold range for pediocin SA-1 production, and 3-fold for lactic acid production (Figure S1, Supplementary Materials).

## 3. Material and Methods

### 3.1. Processing of Fish Discard By-Products

The samples of grenadier (G, *Macrourus* sp.), megrim (Me, *Lepidorhombus boscii*), European hake (Ha, *Merluccius merluccius*), boarfish (Bo, *Capros aper*), and Atlantic horse mackerel (HM, *Trachurus trachurus*) were caught in Atlantic North Ocean by Galician fishing fleets, classified as fish discards, and the death specimens were quickly preserved in ice. The species were immediately processed on the same day of being caught. Fish were manually gutted and headed, and the meat was mechanically separated from bones and skin using a bone separator (Baader 694, Germany). Fish mince was then processed to prepare fish frozen block according the protocol that was described in Blanco et al. [6]. The percentages of heads (H) and skins with bones (SB) that were generated by fish discards processing were in the range of 23.7–53.8% and 3.9–23.5%, respectively.

A sample of SB was collected to evaluate the recovery of gelatin and the rest of SB mixture and H were separately crushed and stored at −18 °C until enzymatic hydrolysis for the production of fish protein hydrolysates (FPHs). A flowchart of the processes that were applied for fish discard valorisation is displayed in Figure S2 (Supplementary Materials).

### 3.2. Gelatin Extraction from SB By-Products

SB from fish discards were treated for gelatin extraction using the methodology that was reported by Sousa et al. [10]. To summarize, the steps for gelatin recovery were: (1) aqueous wash of SB; (2) sequential chemical treatment of portions with NaOH 0.05 M, sulphuric acid 0.02 M, and citric acid 0.05 M solutions; (3) gelatin-water extraction at medium temperature; and, (4) cleaning and deodorization by active charcoal and oven drying of gelatin solutions. Gel strength of gelatins were quantified by texture analysis [13] and levels of proline and hydroyproline were determined by ninhydrin reaction, using an amino acid analyzer according to the method of Moore et al. [50].

### 3.3. Production of FPHs

The hydrolysates of SB and H were prepared in a controlled pH-Stat system with a 5 L glass-reactor including 1 kg of grinded substrates and 2 L of distilled water (S:L ratio of 1:2 *w*/*v*) using 2 M NaOH as alkaline reagent to control pH. The experimental conditions were previously optimized for fish discards by-products (data not shown) and defined, for all cases, as: pH 8.6, stirring at 200 rpm, 60.6 °C, and 1% (*v*/*w*) of Alcalase 2.4 L (Novozymes, Nordisk, Bagsvaerd, Denmark). At the end of the hydrolysis (4 h), the content of the reactors was filtered (100 μm) to remove bones, the liquid hydrolysates were centrifuged (15,000 g, 20 min) to recover oil (adding a step of decantation for 5 min), and the FPHs were quickly heated (90 °C, 15 min) for protease deactivation. Liquid fish peptones were obtained after the sterilisation (121 °C, 15 min) and centrifugation (15,000 g, 20 min) of FPHs (SBP: skin/bone peptone and HP: head peptone).

### 3.4. Chemical Analyses of oils and FPHs

The composition of fatty acids from fish oil was measured by gas chromatography-mass spectrometry after chemical methylation [51]. The basic analysis of FPHs were: (1) total soluble protein [52]; (2) total sugars [53]; (3) total protein as total nitrogen × 6.25 [54]; (4) amino acids content (as quantified by ninhydrin reaction, using an amino acid analyzer (Biochrom 30 series, Biochrom Ltd., Cambridge, UK), according to the method of Moore et al. [50]; and, (5) in vitro digestibility (pepsin method: AOAC Official Method 971.09, following the modifications that were reported by Miller et al. [55]).

Biological activities as antihypertensive and antioxidant (AO) values were quantified in FPHs samples as: (a) in vitro Angiotensin I-converting enzyme (ACE) inhibitory activity (*I_ACE_*) using the protocol that was defined by Estévez et al. [56] and IC_50_ values (protein-hydrolysate concentration that generates 50% of *I_ACE_*), calculated according dose-response modelling [28]; (b) 1,1-Diphenyl-2-picryhydrazyl (DPPH) radical-scavenging ability, following a microplate protocol [57]; (c) ABTS (2,2′-azinobis-(3-ethyl-benzothiazoline-6-sulphonic acid) bleaching method at a microplate scale [57]; and, (d) Crocin bleaching assay also employing an optimised microplate protocol [58]. All of the antihypertensive and AO determinations were done in triplicate, employing FPHs samples at concentration of 1 g/L of soluble protein.

The degree of hydrolysis (*H*, as %) was determined following the pH-Stat method [59] and the equations that are described in a previous report [9]. The kinetics of *H* were finally modelled by the Weibull equation [24]:

(1) $$H=H_{m}\left\{1-\exp\left[-\ln2{\left(\frac{t}{\tau }\right)}^{\beta }\right]\right\}\;\mathrm{with}\;v_{m}=\frac{\beta H_{m}\ln2}{2\tau }$$

where, *H* is the degree of hydrolysis (%); *t* the time of hydrolysis (min); *H_m_* the maximum degree of hydrolysis (%); *β* a parameter that is related with the maximum slope of muscle hydrolysis (dimensionless); *v_m_* the maximum rate of hydrolysis (% min^−1^); and, *τ* is the time required to achieve the semi-maximum degree of hydrolysis (min). The factor of digestion/liquefaction (V_dig_) of raw material to liquid phase was also calculated as the percentage of liquid FPH that is produced relative to the sum of solid raw material and the water and alkalis added for the hydrolysis process.

### 3.5. Fish Peptones from FPHs for Bacterial Culture Media

*Pediococcus acidilactici* NRRL B-5627 was selected to test the capacity of SBP and HP as an organic nitrogen source in low-cost culture media. *Carnobacterium piscicola* CECT 4020 (Spanish Type Culture Collection) was the target bacteria for bacteriocin (Pediocin SA-1) determination. The stock cultures were stored at −80 °C on Man, Rogosa, and Sharpe medium (MRS) with 25% glycerol. Inocula (0.5%, *v*/*v*) consisted of cellular suspensions from 16 h aged in MRS (incubated at 30 °C) and adjusted to an optical density-OD (700 nm) of 0.900.

The composition of the culture media is shown in Table S3 (Supplementary Materials) while employing MRS commercial medium (Pronadisa, Spain) as control. In all cases, the initial pH was adjusted to 7.0 with 5M NaOH and solutions were sterilized at 121 °C for 15 min. Micro-organisms were grown, by duplicate, in 300 mL Erlenmeyer flasks with 180 mL of medium at 30 °C and orbital agitation of 200 rpm. At pre-established times, each culture sample was divided into two aliquots: (1) The first one was processed for the determination of biomass (as dry weight), productions of lactic and acetic acid by HPLC, and the consumption of soluble proteins and reducing sugars accordingly [46,52,60]; (2) The second one was used to extract and determine the antimicrobial activity using *C. piscicola* as an indicator [61,62]. All of the determinations were carried out in duplicate. Growth and metabolite productions were predicted by the logistic equation [63]:

(2) $$P=\frac{P_{m}}{1+\exp\left[2+\frac{4v_{P}}{P_{m}}\left(\lambda_{P}-t\right)\right]}$$

where, *P* is the concentration of the corresponding bioproduction (*X*: biomass, *La*: lactic acid, *BT*: bacteriocin) (in g/L for *X*, *La* and BU/mL for *BT*); t is the time of culture (h); *P_m_* is the maximum concentration of each bioproduction in the asymptotic phase (g/L or BU/mL); *v_P_* is the maximum bioproduction rate (g L^−1^·h^−1^ or BU mL^−1^·h^−1^); and, *λ_P_* is the lag phase of the bioproductions (h).

### 3.6. Numerical and Statistical Analyses

Data fitting procedures and parametric estimations were conducted by the minimisation of the sum of quadratic differences between the observed and model-predicted values, using the non-linear least-squares (quasi-Newton) method that was provided by the macro ‘Solver’ of the Microsoft Excel spreadsheet. Confidence intervals from the parametric estimates (Student’s t test) and the consistence of mathematical models (Fisher’s F test) were evaluated by “SolverAid” macro.

## 4. Conclusions

Starting in 2019, large amounts of new biomasses from fish discards will be generated in European ports following the Landing Obligation guidelines that were issued by the European Commission. In this work, by-products from the mechanical production of fish block mince (heads and skin and bones) were processed to extract gelatin solutions and, by enzymatic proteolysis, oils, fish protein hydrolysates, including bioactive peptides, and fish peptones were produced. As an example of the peptones application, pediocin SA-1, lactic acid, and biomass from *P. acidilactici* were successfully produced in effective-cost media that was formulated with such alternative peptones.

## Figures and Tables

**Table 1:** Characteristics of gelatin isolated from skin and bones (SB) of fish discards. Pro: Proline. OHPro: hydroxyproline. ND: no detected. Errors shown are the confidence intervals for *n* = 2 and α = 0.05.

Fish Discards	Yield (% *w*/*w* Fresh SB)	Pro + OHPro (%)	Strength (Bloom)
**G**	-	-	-
**Bo**	0.23 ± 0.13	>18	ND
**HM**	0.58 ± 0.29	>16	ND
**Ha**	1.71 ± 0.15	>18	58.2 ± 4.4
**Me**	0.88 ± 0.09	>18	30.0 ± 3.2

**Table 2:** Mass balances of the products that were obtained from Alcalase hydrolysis of SB and head (H) of fish discards. Errors shown are the confidence intervals for *n* = 2 and α = 0.05. SB_G: skin/bones of grenadier. SB_Bo: skin/bones of boarfish. SB_Ha: skin/bones of hake. SB_HM: skin/bones of horse mackerel. SB_Me: skin/bones of megrim. H_G: heads of grenadier. H_Bo: heads of boarfish. H_Ha: heads of hake. H_HM: heads of horse mackerel. H_Me: heads of megrim. m_b_: percentage of bones recovered; V_oil_: percentage of oil recovered; V_dig_: percentage of digestion/liquefaction of solid SB or H to the liquid phase; Prs: total soluble protein; TS: total sugars; Dig: digestibility; Pr-tN: total protein as total nitrogen x 6.25.

FPHs	m_b_ (%)	V_oil_ (%)	V_dig_ (%)	Prs (g/L)	Pr-tN (g/L)	TS (g/L)	Dig (%)
**SB_G**	37.4 ± 2.7	-	90.6 ± 3.5	42.2 ± 2.0	43.0 ± 0.8	0.73 ± 0.01	92.1 ± 0.9
**SB_HM**	17.9 ± 1.4	2.35 ± 0.69	86.9 ± 0.8	38.4 ± 0.1	39.3 ± 1.1	0.70 ± 0.01	92.8 ± 0.5
**SB_Bo**	42.4 ± 3.9	0.20 ± 0.04	85.2 ± 0.6	34.2 ± 0.5	34.8 ± 2.2	1.15 ± 0.05	91.7 ± 1.7
**SB_Ha**	22.6 ± 0.3	-	89.5 ± 0.9	33.1 ± 0.5	33.7 ± 1.3	0.59 ± 0.02	93.7 ± 1.0
**SB_Me**	20.6 ± 2.4	1.41 ± 0.11	87.5 ± 0.0	40.4 ± 3.1	41.9 ± 1.1	0.50 ± 0.02	93.9 ± 0.7
**H_G**	21.6 ± 11.5	-	85.8 ± 0.6	29.4 ± 0.7	31.5 ± 2.3	0.83 ± 0.04	92.2 ± 1.4
**H_HM**	14.3 ± 0.3	0.87 ± 0.32	90.5 ± 4.1	27.7 ± 0.9	31.3 ± 1.3	1.06 ± 0.07	90.3 ± 0.5
**H_Bo**	20.3 ± 2.3	0.60 ± 0.20	89.1 ± 2.2	29.1 ± 4.8	34.5 ± 0.7	0.87 ± 0.12	90.1 ± 0.3
**H_Ha**	13.3 ± 0.3	-	88.7 ± 2.6	29.5 ± 0.3	32.8 ± 4.8	0.79 ± 0.08	92.0 ± 0.3
**H_Me**	17.9 ± 1.0	-	84.8 ± 1.6	34.5 ± 1.6	36.4 ± 1.4	0.62 ± 0.06	92.0 ± 0.1

**Table 3:** Kinetic parameters and confidence intervals obtained from Weibull equation (1) modeling the time course of the hydrolysis degree (*H*) of fish discard by-products mediated by alcalase. Determinaton coefficients (R^2^) and p-values are also shown.

FPHs	*H_m_* (%)	α (dimensionless)	*τ* (min)	*v_m_* (% min^−1^)	R^2^	*p*-Values
**SB_Bo**	13.83 ± 0.06	0.537 ± 0.013	6.53 ± 0.20	0.396 ± 0.009	0.993	<0.005
**SB_G**	15.36 ± 0.08	0.664 ± 0.007	28.78 ± 0.35	0.123 ± 0.002	0.999	<0.005
**SB_HM**	19.34 ± 0.06	0.882 ± 0.013	18.87 ± 0.46	0.313 ± 0.009	0.993	<0.005
**SB_Ha**	20.88 ± 0.07	1.062 ± 0.016	33.18 ± 0.35	0.232 ± 0.003	0.998	<0.005
**SB_Me**	21.10 ± 0.14	0.667 ± 0.007	36.78 ± 0.57	0.133 ± 0.002	0.999	<0.005
**H_Bo**	17.55 ± 0.08	0.608 ± 0.013	10.97 ± 0.25	0.337 ± 0.007	0.995	<0.005
**H_G**	18.45 ± 0.06	0.587 ± 0.009	10.71 ± 0.18	0.350 ± 0.005	0.997	<0.005
**H_HM**	21.42 ± 0.08	0.744 ± 0.019	12.93 ± 0.33	0.427 ± 0.010	0.992	<0.005
**H_Ha**	21.86 ± 0.16	0.498 ± 0.016	7.42 ± 0.29	0.509 ± 0.027	0.989	<0.005
**H_Me**	20.41 ± 0.14	0.647 ± 0.012	21.98 ± 0.41	0.208 ± 0.005	0.997	<0.005

**Table 4:** Antioxidant and antihypertensive activities of fish protein hydrolysates (FPHs) obtained from by-products of fish discards. Errors shown are the confidence intervals for *n* = 2 and α = 0.05. ND: not detected; NDe: not determined.

Sample	Antioxidant			Antihypertensive	
**FPHs**	**DPPH (%)**	**ABTS (μg/mL)**	**Crocin (μg/mL)**	**I_ACE_ (%)**	**IC_50_ (μg/mL)**
**SB_G**	34.26 ± 2.85	13.02 ± 2.11	7.45 ± 0.66	57.02 ± 7.10	361.1 ± 39.3
**SB_Bo**	2.29 ± 1.52	3.45 ± 1.88	ND	ND	NDe
**SB_HM**	21.88 ± 4.25	12.13 ± 0.93	4.95 ± 1.87	33.26 ± 27.39	NDe
**SB_Ha**	23.12 ± 1.98	9.45 ± 2.02	3.98 ± 2.67	42.05 ± 2.75	NDe
**SB_Me**	13.25 ± 1.99	6.89 ± 0.88	2.61 ± 1.95	25.41 ± 4.87	NDe
**H_G**	40.28 ± 3.72	16.32 ± 1.72	8.35 ± 0.53	62.18 ± 4.06	195.6 ± 20.7
**H_Bo**	49.12 ± 3.58	25.45 ± 2.12	11.45 ± 0.98	73.77 ± 8.33	178.3 ± 31.3
**H_HM**	25.21 ± 2.09	12.94 ± 1.65	6.53 ± 3.01	45.46 ± 3.97	NDe
**H_Ha**	24.05 ± 2.42	10.55 ± 0.67	5.19 ± 1.74	44.48 ± 8.00	NDe
**H_Me**	10.02 ± 1.52	2.32 ± 1.87	ND	7.71 ± 1.66	NDe

**Table 5:** Numerical values and confidence intervals for parameters derived from logistic equation applied for *P. acidilactici* productions. R^2^ is the determination coefficient among experimental and predicted data. The production yields (*Y_P/Rs_* and *Y_P/Pr_*) are also calculated. NS: not significant.

Parameters	SBP_G	SBP_Ha	SBP_Bo	SBP_HM	SBP_Me	HP_G	HP_Ha	HP_Bo	HP_HM	HP_Me	MRS 1	MRS 2
**Biomass (*X*)**												
***X_m_* (g/L)**	1.74 ± 0.04	1.13 ± 0.04	1.67 ± 0.07	1.25 ± 0.05	1.03 ± 0.06	0.84 ± 0.06	0.86 ± 0.07	0.94 ± 0.07	0.90 ± 0.05	1.08 ± 0.07	1.05 ± 0.08	0.97 ± 0.08
***v_x_*** **(g L^−1^·h^−1^)**	0.15 ± 0.02	0.11 ± 0.02	0.17 ± 0.04	0.14 ± 0.04	0.13 ± 0.06	0.11 ± 0.06	0.10 ± 0.05	0.11 ± 0.05	0.11 ± 0.04	0.11 ± 0.05	0.13 ± 0.06	0.06 ± 0.03
***λ_x_*** **(h)**	4.66 ± 1.09	4.64 ± 1.03	6.32 ± 1.24	5.26 ± 1.33	5.55 ± 2.35	4.15 ± 2.35	4.59 ± 2.34	6.30 ± 2.14	4.22 ± 1.83	4.28 ± 2.34	3.42 ± 2.33	2.98 ± 2.33
***Y_X/Rs_*** **(gX/gRs)**	0.156	0.115	0.171	0.136	0.104	0.088	0.089	0.096	0.136	0.12	0.134	0.131
***Y_X/Pr_*** **(gX/gPr)**	0.942	0.599	0.984	0.798	0.600	0.538	0.564	0.523	0.869	0.650	0.710	0.658
**R^2^**	0.998	0.997	0.996	0.995	0.994	0.981	0.982	0.985	0.989	0.992	0.983	0.982
**Lactic acid (*La*)**												
***La_m_*** **(g/L)**	8.77 ± 0.33	7.57 ± 0.49	6.82 ± 0.49	6.60 ± 0.48	7.67 ± 0.49	6.83 ± 0.49	7.15 ± 0.49	7.72 ± 0.48	7.16 ± 0.83	7.62 ± 0.49	7.43 ± 0.40	7.67 ± 0.40
***v_La_*** **(g L^−1^·h^−1^)**	0.80 ± 0.17	0.67 ± 0.23	0.65 ± 0.26	0.66 ± 0.28	0.76 ± 0.23	0.72 ± 0.23	0.78 ± 0.26	0.80 ± 0.28	0.45 ± 0.23	0.62 ± 0.26	0.78 ± 0.25	0.90 ± 0.25
***λ_La_*** **(h)**	3.69 ± 1.35	3.24 ± 2.21	4.01 ± 2.37	3.49 ± 2.40	3.51 ± 2.21	3.52 ± 2.21	4.15 ± 2.37	4.89 ± 2.40	1.48 (NS)	2.13 ± 2.12	3.58 ± 1.75	4.18 ± 1.75
***Y_La/Rs_*** **(gLa/gRs)**	0.799	0.853	0.773	0.800	0.884	0.765	0.813	0.873	0.825	0.839	0.834	0.841
***Y_La/Pr_*** **(gLa/gPr)**	4.84	4.45	4.44	4.69	5.08	4.67	5.17	4.74	5.29	4.84	4.44	4.24
**R^2^**	0.996	0.988	0.987	0.985	0.987	0.985	0.988	0.992	0.966	0.968	0.992	0.990
**Pediocin (*BT*)**												
***BT_m_*** **(BU/mL)**	209.1 ± 8.3	175.5 ± 8.9	142.4 ± 13.1	176.8 ± 10.5	142.2 ± 20.7	133.1 ± 8.4	127.9 ± 5.6	130.3 ± 9.3	151.8 ± 12.2	146.7 ± 11.2	204.5 ± 10.2	220.7 ± 13.6
***v_BT_*** **(BU mL^−1^·h^−1^)**	14.7 ± 2.7	10.3 ± 2.0	5.79 ± 1.15	8.6 ± 1.5	6.13 ± 2.26	10.0 ± 2.9	9.30 ± 1.81	7.81 ± 2.09	8.09 ± 2.27	8.94 ± 2.72	13.6 ± 2.9	12.2 ± 2.9
***λ_BT_*** **(h)**	6.7 ± 1.3	7.60 ± 1.70	11.4 ± 2.4	10.7 ± 1.8	9.88 ± 4.22	9.36 ± 1.99	9.90 ± 1.36	10.1 ± 2.3	8.25 ± 2.63	8.02 ± 2.53	7.68 ± 1.66	6.07 ± 2.14
***Y_BT/Rs_*** **(BU/mgRs)**	18.80	18.92	15.30	20.15	16.14	13.71	13.96	14.71	17.13	15.42	22.29	23.63
***Y_BT/Pr_*** **(BU/mgPr)**	113.70	98.76	87.79	118.10	92.67	83.66	88.71	79.93	109.71	89.02	118.54	119.04
**R^2^**	0.997	0.996	0.995	0.997	0.983	0.994	0.997	0.994	0.992	0.992	0.996	0.994

## Supplementary Materials

The following are available online at https://www.mdpi.com/1660-3397/17/3/139/s1, Table S1: Fatty acids content from fish oils recovered from different by-products of SB and H fish discards; Table S2: Amino acids content of FPHs produced from SB and H by-products from fish discards; Table S3: Composition of the culture media used for the fermentation of *P. acidilactici*; Figure S1. Costs of the metabolites generated by *P. acidilactici* growing in MRS and low-cost media; Figure S2: Flowchart of fish discards valorization.
