# Supplementary material for: Production of Valuable Compounds and Bioactive Metabolites from By-Products of Fish Discards Using Chemical Processing, Enzymatic Hydrolysis, and Bacterial Fermentation
Source: Mar Drugs. 2019 Feb 27;17(3):139. doi: 10.3390/md17030139 (PMC6470541; doi:10.3390/md17030139)
Supplement: Supplementary file 1 [file marinedrugs-17-00139-s001.pdf]

SUPPLEMENTARY MATERIAL

Table S1. Fatty acids (as %) content from fish oils recovered from different by-products of SB and H fish discards. Acronyms are defined in Table 2. Errors shown are the confidence intervals for $n = 2$ and $\alpha = 0.05$.

Formula	Fatty Acids	SB_HM	H_HM	H_Bo	SB_Me
**C14:0**	Myristic acid	5.60 ± 0.41	5.59 ± 0.24	7.61 ± 0.35	3.749 ± 0.20
**C14:1**	Myristoleic acid	0.37 ± 0.06	0.12 ± 0.03	0.08 ± 0.04	0.20 ± 0.00
**C15:0**	Pentadecanoic acid	0.43 ± 0.08	0.54 ± 0.11	0.53 ± 0.17	0.58 ± 0.05
**C15:1**	Pentadecenoic acid	9.26 ± 0.38	9.74 ± 0.61	8.43 ± 0.98	8.18 ± 0.45
**C16:0**	Palmitic acid	14.30 ± 0.62	15.43 ± 0.98	12.92 ± 1.03	12.63 ± 0.39
**C16:1n7c**	Palmitoleic acid	8.25 ± 0.08	9.14 ± 0.51	8.61 ± 0.59	15.18 ± 0.62
**C17:0**	Heptadecanoic acid	0.44 ± 0.01	0.48 ± 0.04	0.33 ± 0.02	0.38 ± 0.02
**C17:1**	Heptadecanoleic acid	0.42 ± 0.03	0.51 ± 0.08	0.43 ± 0.05	0.53 ± 0.03
**C18:0**	Stearic acid	3.16 ± 0.25	3.02 ± 0.21	2.48 ± 0.29	2.22 ± 0.18
**C18:1n9c,t**	Oleic acid	17.95 ± 0.87	17.52 ± 0.69	16.12 ± 1.17	19.39 ± 0.75
**C18:2n6c,t**	Linoleic acid	1.67 ± 0.26	1.71 ± 0.31	1.15 ± 0.15	2.18 ± 0.08
**C20:0**	Arachidic acid	0.33 ± 0.02	0.40 ± 0.01	0.45 ± 0.04	0.15 ± 0.02
**C18:3n3**	Linolenic acid	0.54 ± 0.15	0.63 ± 0.10	0.40 ± 0.05	0.61 ± 0.06
**C18:4n3**	Stearidonic acid	-	-	-	-
**C18:3n6**	-Linolenic acid	0.75 ± 0.10	0.88 ± 0.07	0.56 ± 0.09	0.11 ± 0.11
**C20:1n9**	Eicosenoic acid	4.32 ± 0.39	3.17 ± 0.08	8.08 ± 0.52	1.68 ± 0.31
**C20:4n3**	Eicosatetraenoic acid	-	-	-	-
**C20:3n6**	Dihomo- -linolenic acid (DGLA)	1.55 ± 0.14	1.14 ± 0.10	2.81 ± 0.19	0.59 ± 0.15
**C20:4n6**	Arachidonic acid	0.54 ± 0.17	0.77 ± 0.09	0.52 ± 0.13	1.41 ± 0.28
**C20:5n3**	Eicosapentaenoic acid (EPA)	7.07 ± 0.51	8.20 ± 0.29	4.13 ± 0.35	10.10 ± 0.45
**C21:4n3**	Heneicosatetraenoic acid	1.89 ± 0.15	2.50 ± 0.41	2.31 ± 0.18	1.84 ± 0.25
**C21:5n3**	Heneicosapentaenoic acid (HPA)	-	-	-	-
**C22:0**	Docosanoic acid	0.15 ± 0.02	1.65 ± 0.11	4.08 ± 0.31	0.24 ± 0.13
**C22:2n6**	Docosadienoic acid	4.07 ± 0.34	0.32 ± 0.07	0.36 ± 0.02	0.30 ± 0.05
**C22:5n6**	Docosapentaenoic acid	-	-	-	-
**C22:5n3**	Docosapentaenoic acid (DPA)	-	-	-	-
**C22:6n3**	Docosahexaenoic acid (DHA)	9.60 ± 0.60	11.96 ± 0.50	8.63 ± 0.81	16.01 ± 0.35
	Others	5.51 ± 0.45	4.61 ± 0.81	9.00 ± 0.47	1.71 ± 0.40
	**DHA + EPA (%)**	16.67	20.16	12.76	26.11
	**r: omega-3/omega-6**	2.23	4.84	2.87	5.84

Table S2. Amino acids content of FPHs (% or g/100 g total amino acids) produced from SB and H by-products from fish discards. OHPro:hydroxyproline. Errors shown are the confidence intervals for $n = 2$ and $\alpha = 0.05$.

Amino Acids	SB_G	SB_Bo	SB_Ha	SB_HM	SB_Me	H_G	H_Bo	H_Ha	H_HM	H_Me
**Asp**	9.46 ± 0.10	8.86 ± 0.02	8.84 ± 0.52	7.98 ± 0.22	7.61 ± 0.78	9.89 ± 0.06	10.04 ± 0.15	9.90 ± 0.01	9.45 ± 0.32	9.54 ± 0.57
**Thr**	3.75 ± 0.04	4.12 ± 0.03	4.02 ± 0.12	4.00 ± 0.02	4.13 ± 0.42	4.08 ± 0.04	4.30 ± 0.04	4.42 ± 0.15	4.29 ± 0.08	4.21 ± 0.28
**Ser**	6.08 ± 0.32	4.69 ± 0.08	5.82 ± 0.05	5.85 ± 0.07	5.39 ± 0.44	5.32 ± 0.23	5.16 ± 0.61	5.36 ± 0.10	5.34 ± 0.15	5.28 ± 0.60
**Glu**	13.06 ± 0.31	12.41 ± 0.11	12.45 ± 0.04	12.36 ± 0.33	11.84 ± 0.69	13.82 ± 0.05	15.36 ± 0.46	14.03 ± 0.25	14.12 ± 0.38	16.49 ± 0.79
**Gly**	14.76 ± 0.06	11.37 ± 0.10	16.23 ± 0.44	15.94 ± 0.32	16.12 ± 0.10	8.52 ± 0.06	7.11 ± 0.02	8.27 ± 0.11	6.73 ± 0.23	6.90 ± 0.73
**Ala**	8.94 ± 0.13	7.85 ± 0.06	9.26 ± 0.08	9.01 ± 0.15	8.65 ± 0.63	7.00 ± 0.13	6.37 ± 0.14	7.03 ± 0.21	6.57 ± 0.05	6.51 ± 0.75
**Cys**	0.31 ± 0.02	0.44 ± 0.03	0.25 ± 0.01	0.29 ± 0.06	0.33 ± 0.01	0.45 ± 0.02	0.39 ± 0.01	0.40 ± 0.03	0.45 ± 0.00	0.33 ± 0.12
**Val**	3.17 ± 0.07	4.39 ± 0.05	3.34 ± 0.12	3.67 ± 0.37	3.72 ± 0.31	4.28 ± 0.02	4.23 ± 0.03	4.38 ± 0.05	4.39 ± 0.10	4.17 ± 0.09
**Met**	3.09 ± 0.06	3.11 ± 0.04	2.60 ± 0.01	2.12 ± 0.08	2.00 ± 0.17	3.65 ± 0.05	3.78 ± 0.25	3.41 ± 0.23	3.93 ± 0.64	3.45 ± 0.53
**Ile**	1.95 ± 0.01	3.44 ± 0.05	1.80 ± 0.03	2.00 ± 0.13	2.05 ± 0.04	3.32 ± 0.04	3.22 ± 0.20	3.41 ± 0.08	3.53 ± 0.35	3.91 ± 1.40
**Leu**	6.03 ± 0.01	6.57 ± 0.01	5.55 ± 0.12	5.68 ± 0.09	5.62 ± 0.07	7.43 ± 0.03	6.89 ± 0.25	7.45 ± 0.08	7.12 ± 0.34	7.80 ± 0.81
**Tyr**	2.15 ± 0.10	2.69 ± 0.02	2.42 ± 0.09	2.81 ± 0.12	2.91 ± 0.05	3.28 ± 0.08	4.29 ± 1.35	3.33 ± 0.05	4.78 ± 0.78	3.72 ± 0.62
**Phe**	4.29 ± 0.21	4.12 ± 0.02	3.67 ± 0.09	4.05 ± 0.40	4.63 ± 0.70	4.55 ± 0.06	4.10 ± 0.34	4.40 ± 0.20	4.38 ± 0.31	3.28 ± 0.30
**His**	2.03 ± 0.36	1.35 ± 0.12	2.18 ± 0.09	2.29 ± 0.24	2.44 ± 0.51	1.55 ± 0.08	2.39 ± 0.26	1.72 ± 0.12	2.57 ± 0.07	2.21 ± 0.29
**Lys**	5.80 ± 0.25	6.33 ± 0.04	5.37 ± 0.70	5.94 ± 0.48	6.11 ± 0.77	7.08 ± 0.07	7.53 ± 0.18	7.25 ± 0.18	7.48 ± 0.51	6.92 ± 0.25
**Arg**	6.42 ± 0.09	6.08 ± 0.02	6.34 ± 0.06	6.47 ± 0.27	6.74 ± 0.76	6.55 ± 0.06	6.49 ± 0.34	6.31 ± 0.08	6.76 ± 0.31	6.24 ± 0.13
**OHPro**	2.55 ± 0.01	6.40 ± 0.04	3.79 ± 0.19	3.60 ± 0.42	3.64 ± 0.47	4.82 ± 0.25	3.49 ± 0.47	4.15 ± 0.73	3.50 ± 0.42	4.17 ± 0.17
**Pro**	6.18 ± 0.04	5.80 ± 0.07	6.06 ± 0.11	5.93 ± 0.14	6.08 ± 0.38	4.42 ± 0.26	4.85 ± 0.52	4.77 ± 0.33	4.62 ± 0.13	4.87 ± 0.13
**Pr (Σaa) (g/L)**	42.82 ± 2.79	35.82 ± 2.79	40.83 ± 4.36	39.84 ± 2.59	42.69 ± 0.60	34.70 ± 0.82	35.23 ± 0.92	34.03 ± 3.92	34.49 ± 0.41	37.22 ± 0.44

Table S3. Composition of the culture media (in g/L) used for the fermentation of *P. acidilactici*. SBP_G: culture medium formulated with SB peptone from grenadier. SBP_Ha: culture medium formulated with SB peptone from hake. SBP_Bo: culture medium formulated with SB peptone from boarfish. SBP_HM: culture medium formulated with SB peptone from horse mackerel. SBP_Me: culture medium formulated with SB peptone from megrim. HP_G: culture medium formulated with H peptone from grenadier. HP_Ha: culture medium formulated with H from hake. HP_Bo: culture medium formulated with H peptone from boarfish. HP_HM: culture medium formulated with H peptone from horse mackerel. HP_Me: culture medium formulated with H peptone from megrim.

	**SBP_G**	**SBP_Ha**	**SBP_Bo**	**SBP_HM**	**SBP_Me**	**HP_G**	**HP_Ha**	**HP_Bo**	**HP_HM**	**HP_Me**	**MRS**
Glucose	20	20	20	20	20	20	20	20	20	20	20
Yeast extract	4	4	4	4	4	4	4	4	4	4	4
Sodium acetate	5	5	5	5	5	5	5	5	5	5	5
Ammonium citrate	2	2	2	2	2	2	2	2	2	2	2
K_2HPO_4	2	2	2	2	2	2	2	2	2	2	2
$MgSO_4$	0.2	0.2	0.2	0.2	0.2	0.2	0.2	0.2	0.2	0.2	0.2
$MnSO_4$	0.05	0.05	0.05	0.05	0.05	0.05	0.05	0.05	0.05	0.05	0.05
Tween 80	1	1	1	1	1	1	1	1	1	1	1
Meat extract	-	-	-	-	-	-	-	-	-	-	8
Bactopeptone	-	-	-	-	-	-	-	-	-	-	10
SB or H Peptone*	10	10	10	10	10	10	10	10	10	10	-

*Soluble proteins (as Lowry-method) at 10 g/L in the final media.

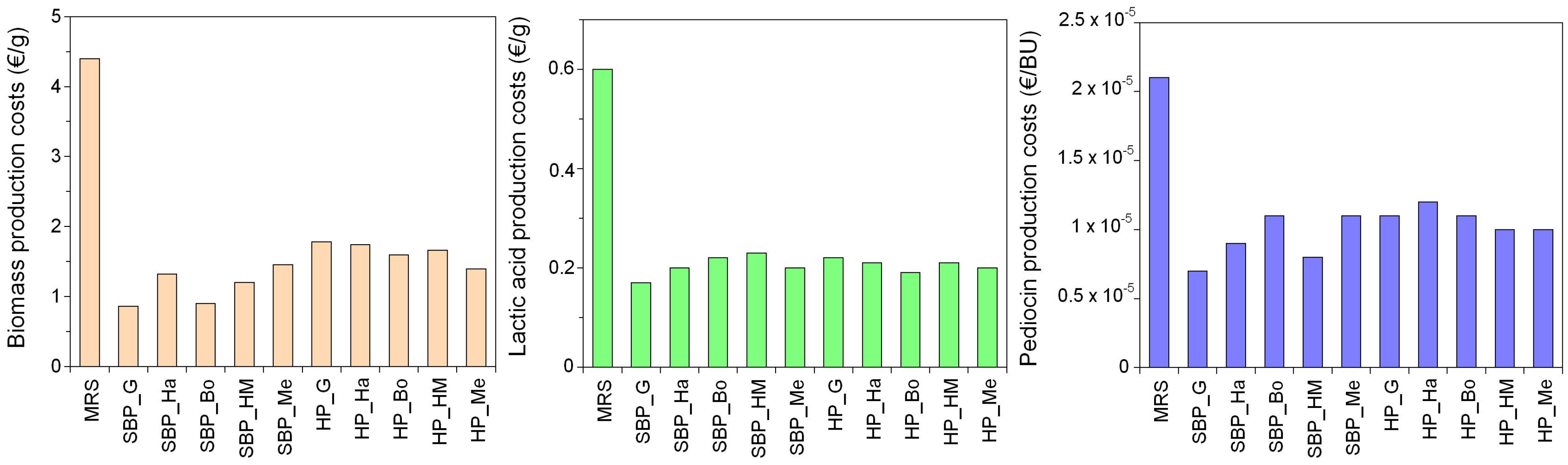

Figure S1. Costs of the metabolites generated by *P. acidilactici* growing in MRS and low-cost media.

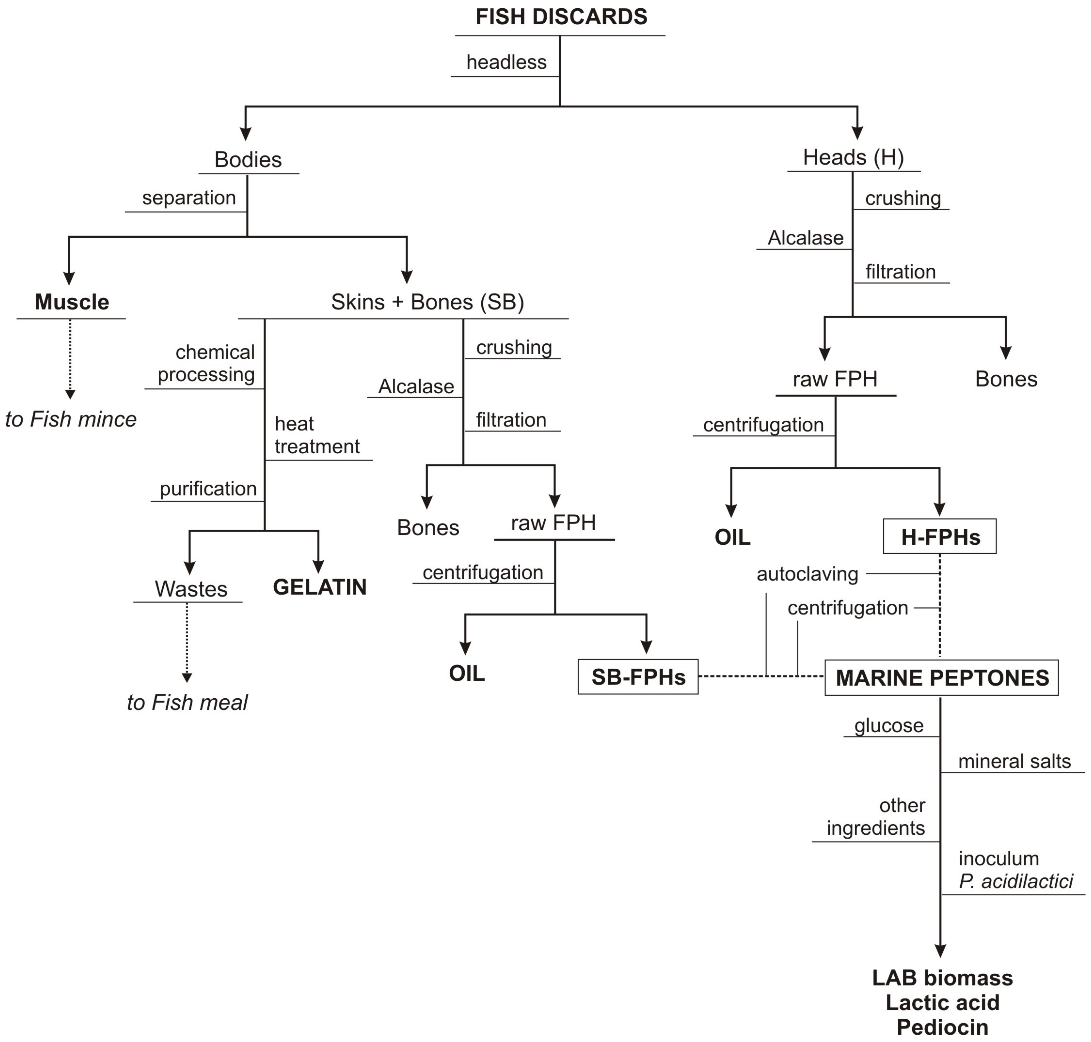

Figure S2. Flowchart of fish discards valorisation.
